# Targeting LncRNA EPIC1 to inhibit human colon cancer cell progression

**DOI:** 10.18632/aging.103790

**Published:** 2020-11-07

**Authors:** Qiong Wu, Jue Wei, Chen Zhao, Shihao Xiang, Min Shi, Yugang Wang

**Affiliations:** 1 Department of Gastroenterology, Tongren Hospital, Shanghai Jiao Tong University School of Medicine, Shanghai, China

**Keywords:** colon cancer, LncRNA, Lnc-EPIC1, MYC

## Abstract

Long non-coding RNA EPIC1 (Lnc-EPIC1) binds MYC protein, which is essential for MYC function and expression of MYC target genes. The current study tested its expression and potential functions in human colon cancer cells. We show that Lnc-EPIC1 expression is elevated in human colon cancer tissues and primary human colon cancer cells. Whereas its expression is relatively low in normal colon tissues and colon epithelial cells. In the primary human colon cancer cells, Lnc-EPIC1 siRNA largely inhibited cancer cell growth, proliferation, migration and invasion. Further, Lnc-EPIC1 silencing induced significant apoptosis activation in colon cancer cells. Conversely, ectopic overexpression of Lnc-EPIC1 augmented colon cancer cell growth, proliferation, migration and invasion. RNA-immunoprecipitation and RNA pull-down results confirmed that Lnc-EPIC1 directly binds MYC protein in colon cancer cells. MYC target proteins, including cyclin A, cyclin D and CDK9, were downregulated with Lnc-EPIC1 silencing, but upregulated after Lnc-EPIC1 overexpression in colon cancer cells. Further Lnc-EPIC1 silencing or overexpression failed to alter functions of MYC-knockout colon cancer cells. Collectively, overexpressed Lnc-EPIC1 is important for the progression of human colon cancer cells.

## INTRODUCTION

Colon cancer is one of the leading causes of cancer-related human mortalities around the word [1–4]. The current treatment options for colon cancer include surgery, chemotherapy and molecularly-targeted therapy [1, 2]. Over the past decades, significant advances have been achieved in basic research and medical treatments for colon cancer, yet the overall survival for the advanced, metastatic and recurrent colon cancer is still below 50% [5, 6]. A large proportion of colon cancer patients are resistant to the current treatments [5, 6]. One possible reason is that the underlying mechanisms of colon cancer development, tumorigenesis and progression are still largely elusive.

Studies have shown that 95% of the genome transcripts are non-coding RNAs (ncRNAs), including microRNA (miRNAs), long noncoding RNAs (LncRNAs), and circular RNAs (circRNAs) [7, 8]. LncRNAs are transcripts over 200 nucleotides long but without protein coding potential [9, 10]. LncRNAs participates in all cellular and physiological behaviors. These include embryo development, immune regulation and cancer progression [9, 10]. LncRNAs are dysregulated in colon cancer and other human cancers [8–10], emerging as possible diagnosis markers and therapeutic targets [8, 10–12].

Wang et al., have recently discovered LncRNA EPIC1 (ENSG00000224271, “Lnc-EPIC1”) directly associates with the oncogenic protein MYC (c-MYC) [13]. MYC directly binds LncRNA EPIC1 at 129-283 nucleotide region [13], which is essential for MYC’s function and expression of MYC target genes [13]. Lnc-EPIC1 inhibition or silencing decreased the occupancy of MYC to its target genes (*CDC20*, *CDC45* and *cyclin A* etc) [13], thereby downregulating their expression [13]. The results of the present study show that Lnc-EPIC1 is overexpressed in human colon cancer and associated with colon cancer cell progression.

## RESULTS

### Lnc-EPIC1 is overexpression in human colon cancer tissues and cells

**Figure 1:**
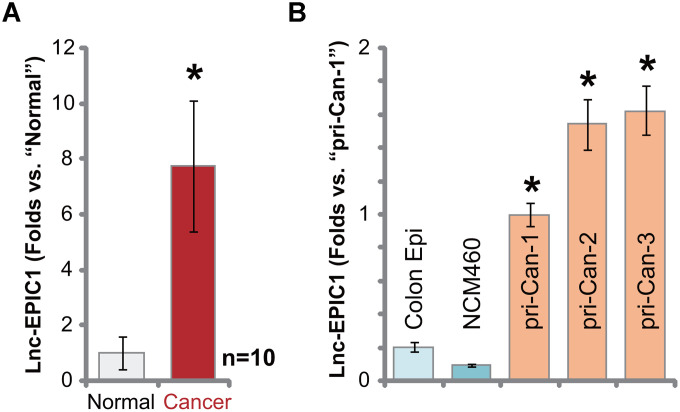
**Lnc-EPIC1 is elevated in human colon cancer tissues and cells.** Expression of Lnc-EPIC1 in the primary colon cancer tissues (“Cancer”) and matched surrounding normal colon mucosa epithelial tissues (“Normal”) of ten different human patients (n=10) was tested by qPCR (**A**); The relative expression of Lnc-EPIC1 in primary human colon cancer cells, “pri-Can-1/-2/-3”, NCM460 cells or primary human colon epithelial cells (“Colon Epi”) was shown (**B**). Data presented as mean ± standard deviation (SD). * *p*< 0.05 vs. “Normal” tissues or “Colon Epi” cells. Experiments in this figure were repeated three times.

Ten different primary colon cancer tissues (“Cancer”) along with the paired surrounding normal colon mucosa epithelial tissues (“Normal”) were collected and incubated with tissue lysis buffer. Total RNA was subjected to qPCR analyses. Results in Figure 1A demonstrated that Lnc-EPIC1 expression increased over seven folds in colon cancer tissues, when compared to it in the normal colon tissues. Elevation of Lnc-EPIC1 expression was detected as well in primary human colon cancer cells (Figure 1B). The primary cancer cells were derived from different human patients, pri-Can-1/-2/-3 (from Dr. Xu at Tong-ren Hospital [14]). Lnc-EPIC1 expression level is relatively low in NCM460 colon mucosa epithelial cells and primary human colon epithelial cells (“Colon Epi”) (Figure 1B). These results show that Lnc-EPIC1 is upregulated in human colon cancer tissues and cells.

### Lnc-EPIC1 siRNA inhibits colon cancer cell growth, proliferation, migration and invasion

**Figure 2:**
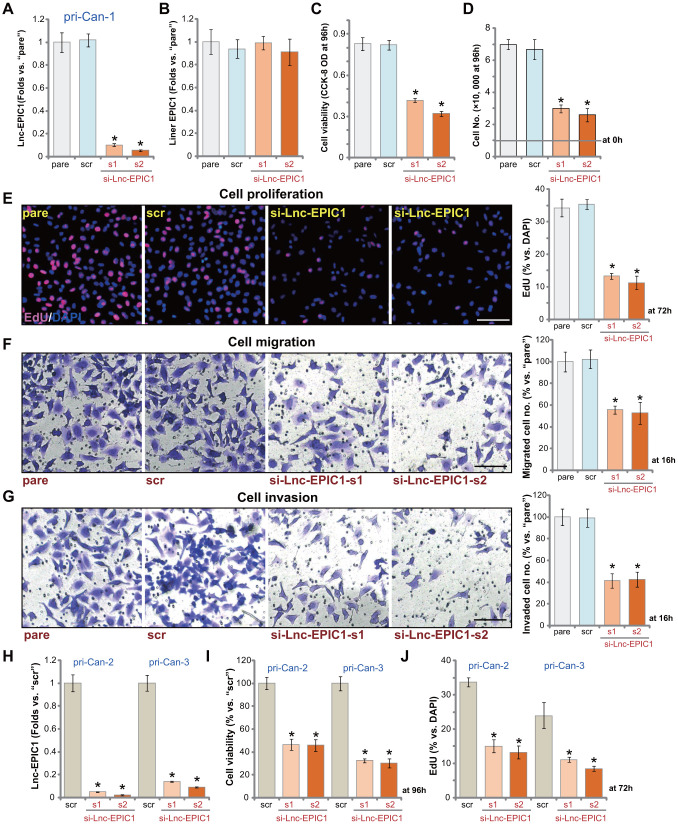
**Lnc-EPIC1 siRNA inhibits colon cancer cell growth, proliferation, migration and invasion.** The primary human colon cancer cells, pri-Can-1/-2/-3, were transfected with scramble control siRNA (“scr”, 500 nM) or the applied Lnc-EPIC1 siRNA (si-Lnc-EPIC1-s1/si-Lnc-EPIC1-s2, 500 nM), cells were further cultured for 48h, and expression of Lnc-EPIC1 and *linear EPIC1* was tested by qPCR assays (**A**, **B**, **H**); Cells were further cultured for applied time periods, cell viability was examined by CCK-8 assays (**C**, **I**); Cell growth and proliferation were examined by cell counting assay (**D**) and nuclear EdU staining (**E**, **J**). Cell migration and invasion were tested by “Transwell” (**F**) and “Matrigel Transwell” (**G**) assays, respectively. For EdU studies, ten random views of each treatment were included to calculate the average EdU ratio (% vs. DAPI, same for all Figures). For “Transwell”/“Martial Transwell” assays, in each condition ten random views were included to calculate the average number of migrated/invaded cells (same for all Figures). The exact same number of viable colon cancer cells with different genetic modifications were initially seeded into each well or each dish (at 0h, same for all Figures). “pare” stands for the parental control cells (same for all Figures). Data presented as mean ± standard deviation (SD). * *p*< 0.05 vs. “scr” cells. Experiments in this figure were repeated five times. Bar=100 μm (**E**–**G**).

In order to test the potential effect of Lnc-EPIC1 on colon cancer cell behaviors, siRNA strategy was applied to knockdown Lnc-EPIC1. As described, two different Lnc-EPIC1 siRNAs, si-Lnc-EPIC1-s1 and si-Lnc-EPIC1-s2 (see previous studies [15, 16]), were transfected to primary human colon cancers (“pri-Can-1”). Each of the two resulted in over 90% downregulation of Lnc-EPIC1 expression (Figure 2A). The applied Lnc-EPIC1 siRNAs failed to alter expression of *liner EPIC1* (Figure 2B). Testing cell viability, using a CCK-8 assay kit, demonstrated that Lnc-EPIC1 silencing by targeted siRNAs decreased viability of colon cancer cells (Figure 2C). Cell counting assay results, Figure 2D, confirmed that Lnc-EPIC1 siRNAs inhibited colon cancer cell growth. The percentage of nuclei with positive EdU staining was significantly decreased in Lnc-EPIC1-silenced colon cancer cells, indicating proliferation inhibition with Lnc-EPIC1 silencing (Figure 2E). To test cell migration and invasion *in vitro*, “Transwell” and “Matrigel Transwell” assays were performed. Results demonstrated that Lnc-EPIC1 silencing resulted in over 50-60% reduction of colon cancer cell migration (Figure 2F) and invasion (Figure 2G). The “Transwell” assays were performed at 16h after incubation to exclude the possible influence of cell proliferation and growth.

In the primary human colon cancer cells that were derived from two other colon cancer patients, pri-Can-2 and pri-Can-3, transfection of si-Lnc-EPIC1-s1 or si-Lnc-EPIC1-s2 resulted in over 90% reduction of Lnc-EPIC1 expression (Figure 2H). Lnc-EPIC1 silencing inhibited cell viability (CCK-8 OD, Figure 2I) and proliferation (counting EdU-positive nuclei, Figure 2J). The scramble control siRNA (“scr”) failed to alter Lnc-EPIC1 expression and cancer cell behaviors (Figure 2A–2J). These results clearly demonstrated that Lnc-EPIC1 silencing by targeted siRNAs inhibited human colon cancer cell growth, proliferation, migration and invasion.

### Lnc-EPIC1 siRNA provokes apoptosis in colon cancer cells

**Figure 3:**
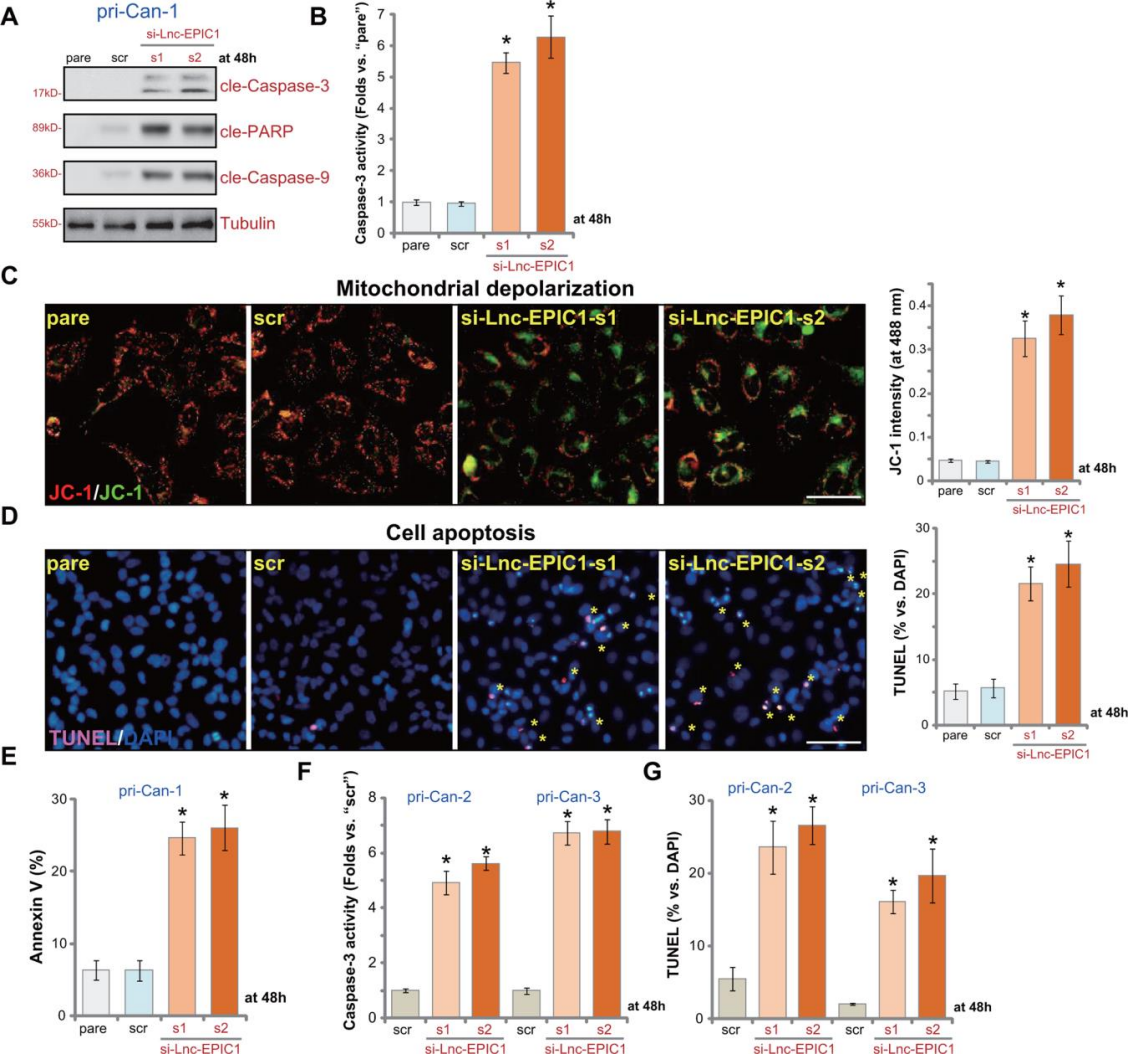
**Lnc-EPIC1 siRNA provokes apoptosis in colon cancer cells.** The primary human colon cancer cells, pri-Can-1/-2/-3, were transfected with scramble control siRNA (“scr”, 500 nM) or the applied Lnc-EPIC1 siRNA (si-Lnc-EPIC1-s1/si-Lnc-EPIC1-s2, 500 nM, for 48h), cells were further cultured for additional 48h, expression of listed proteins in total cell lysates was tested by Western blotting assays (**A**), the relative caspase-3 activity was tested (**B**, **F**), with mitochondrial depolarization examined by JC-1 staining (**C**); Cell apoptosis was examined and quantified by nuclear TUNEL staining (**D**, **G**) and Annexin V FACS (**E**). Data presented as mean ± standard deviation (SD). * *p*< 0.05 vs. “scr” cells. Experiments in this figure were repeated five times. Bar=100 μm (**C**, **D**).

In human cancer cells inhibition of proliferation and growth can induce cell apoptosis. As shown in pri-Can-1 cells with si-Lnc-EPIC1-s1/2 (Figure 2), cleavages of caspase-3, caspase-9 and PARP were detected (Figure 3A). Furthermore, Lnc-EPIC1 silencing resulted in 5-6 folds of increase of caspase-3 activity in colon cancer cells (Figure 3B). Lnc-EPIC1 knockdown in pri-Can-1 cells induced mitochondrial depolarization, the latter was evidenced by accumulation of JC-1 green monomers (Figure 3C). Furthermore, with Lnc-EPIC1 silencing the ratio of TUNEL-positive nuclei (% vs. DAPI) was significantly increased (Figure 3D). Additionally, the applied Lnc-EPIC1 siRNAs resulted in significantly increased number of Annexin V-positive pri-Can-1 cells (Figure 3E). In other primary human colon cancer cells, pri-Can-2 and pri-Can-3, si-Lnc-EPIC1-s1/2 similarly increased Caspase-3 activity (Figure 3F) and TUNEL-positive nuclei ratio (Figure 3G). These results show that Lnc-EPIC1 siRNA provoked apoptosis activation in primary human colon cancer cells. The scramble control siRNA was ineffective on cell apoptosis (Figure 3A–3G).

### Ectopic overexpression of Lnc-EPIC1 promotes colon cancer cell progression *in vitro*

**Figure 4:**
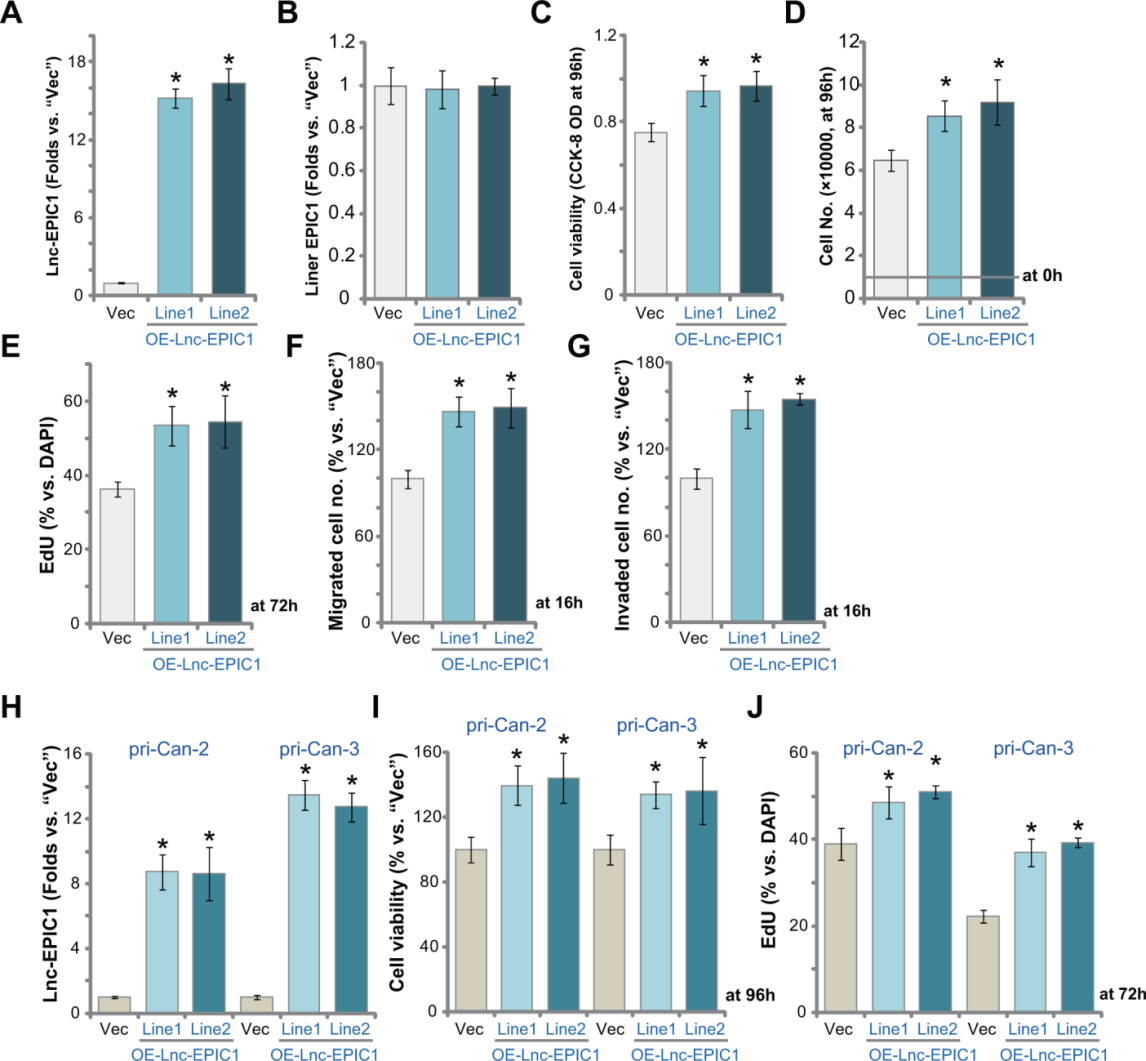
**Ectopic overexpression of Lnc-EPIC1 promotes colon cancer cell progression *in vitro.*** The stable human colon cancer cells pri-Can-1/-2/-3, with empty vector (“Vec”) or the Lnc-EPIC1-expression construct (“OE-Lnc-EPIC1-Line1/2”, indicating two stable lines) were cultured for applied time periods, expression of Lnc-EPIC1 and *linear EPIC1* was tested by qPCR assays (**A**, **B**, **H**); Cell viability was examined by CCK-8 assays (**C**, **I**); Cell growth and proliferation were examined by cell counting assay (**D**) and nuclear EdU staining (**E**, **J**), respectively. Cell migration and invasion were tested by “Transwell” assay (**F**) and “Matrigel Transwell” assay (**G**), respectively. Results were quantified. Data presented as mean ± standard deviation (SD). * *p*< 0.05 vs. “Vec” cells. Experiments in this figure were repeated five times.

We hypothesized that forced overexpression of Lnc-EPIC1 could further promote colon cancer cell progression *in vitro*. To support our hypothesis a Lnc-EPIC1-expression vector (provided by Dr. Sun [17]) was transduced to pri-Can-1 colon cancer cells. Cells were further selected by puromycin to establish stable cell lines: OE-Lnc-EPIC1-Line1 and OE-Lnc-EPIC1-Line2. As compared to the vector control cells (“Vec”), the mature Lnc-EPIC1 expression increased over 15 folds in OE-Lnc-EPIC1 cells (Figure 4A), whereas the *liner EPIC1* levels were unchanged (Figure 4B). Cell viability, the CCK-8 OD, was increased in primary cancer cells with Lnc-EPIC1 overexpression (Figure 4C). The cell counting assay results, Figure 4D, confirmed that OE-Lnc-EPIC1 colon cancer cells grew significantly faster than the vector control cells.

In pri-Can-1 colon cancer cells ectopic overexpression of Lnc-EPIC1 increased cell proliferation (EdU incorporation, Figure 4E). Cell migration and invasion were augmented as well, the two were tested by “Transwell” (Figure 4G) and “Matrigel Transwell” (Figure 4H) assays, respectively. In pri-Can-2 and pri-Can-3 colon cancer cells, the Lnc-EPIC1-expression vector resulted in significant increase of Lnc-EPIC1 expression (Figure 4H). It significantly increased cell viability (CCK-8 OD, Figure 4I) and proliferation (EdU ratio, Figure 4J). Thus, ectopic overexpression of Lnc-EPIC1 further promoted colon cancer cell growth, proliferation, migration and invasion.

### Lnc-EPIC1-MYC binding promotes colon cancer cell progression

**Figure 5:**
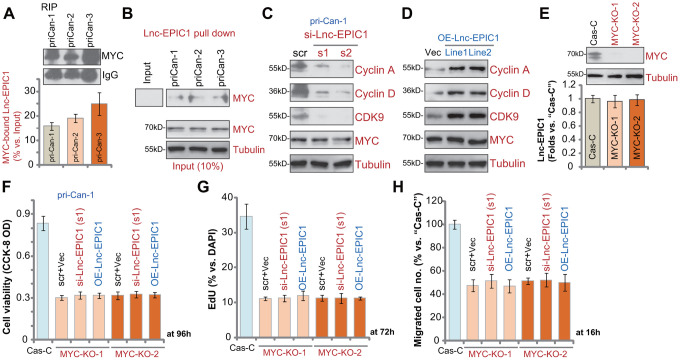
**Lnc-EPIC1-MYC binding promotes colon cancer cell progression.** The qPCR analyses of Lnc-EPIC1 enriched in the MYC protein in primary human colon cancer cells, pri-Can-1/-2/-3 (**A**). Western blotting analyses of the MYC protein retrieved by *in-vitro*-transcribed Lnc-EPIC1 in primary human colon cancer cells, pri-Can-1/-2/-3 (**B**). The primary human colon cancer cells, pri-Can-1, were transfected with scramble control siRNA (“scr”, 500 nM) or the applied Lnc-EPIC1 siRNA (si-Lnc-EPIC1-s1/si-Lnc-EPIC1-s2, 500 nM, for 48h), expression of listed proteins was shown (**C**). Expression of the listed proteins in pri-Can-1 cells with empty vector (“Vec”) or the Lnc-EPIC1-expression construct (“OE-Lnc-EPIC1-Line1/2”) was shown (**D**). Expression of MYC protein and Lnc-EPIC1 in stable pri-Can-1 cells with the CRISPR/Cas9 MYC-KO construct (“MYC-KO-1/MYC-KO-2”, two constructs with different sgRNAs) or the empty vector (“Cas-C”) was shown (**E**); Cells were further transfected with si-Lnc-EPIC1-s1 (500 nM, for 48h) or Lnc-EPIC1-expression construct (“OE-Lnc-EPIC1”, for 48h), and further cultured for the applied time periods, cell viability, proliferation and migration were tested by CCK-8 viability (**F**), nuclear EdU staining (**G**) and “Transwell” (**H**) assays, respectively, and results were quantified. Data presented as mean ± standard deviation (SD). Experiments in this figure were repeated five times.

To test whether there is a direct association between MYC protein and Lnc-EPIC1 in human colon cancer cells, RNA-immunoprecipitation (RIP) experiments were performed. In primary human colon cancer cells, pri-Can-1/-2/-3, the MYC (c-MYC) protein immunoprecipitated with Lnc-EPIC1 (Figure 5A). Furthermore, by applying a RNA pull-down assay, we show that the biotin-labeled full-length Lnc-EPIC1 associated with MYC protein in pri-Can-1/-2/-3 cells (Figure 5B). These results indicated that MYC directly binds Lnc-EPIC1 in colon cancer cells. To indicate the functional consequence of MYC-Lnc-EPIC1 binding, we show that expression of MYC targets, cyclin A, cyclin D and CDK9, was downregulated by Lnc-EPIC1 siRNA in pri-Can-1 cells (Figure 5C). Conversely, the three were elevated in Lnc-EPIC1-overexpressed pri-

Can-1 cells (Figure 5D). MYC was unchanged in Lnc-EPIC1-silenced or Lnc-EPIC1-overexpressed cells (Figure 5C, 5D). These results suggested that MYC-Lnc-EPIC1 association is essential for MYC function in human colon cancer cells.

If Lnc-EPIC1-induced colon cancer cell progression is due to association with MYC, it should be ineffective in MYC-depleted cells. To test this hypothesis, two different CRISPR/Cas9 MYC-KO constructs (provided by Dr. Sun at Yangzhou University Medical School [17]) were individually transduced to pri-Can-1 colon cancer cells. Stable cells were established, showing completely depleted MYC (Figure 5E). Lnc-EPIC1 expression was unchanged in MYC-KO cells (Figure 5E). CRISPR/Cas9-induced MYC KO significantly inhibited colon cancer cell survival (Figure 5F), proliferation (Figure 5G) and migration (Figure 5H). Importantly, Lnc-EPIC1 siRNA (by si-Lnc-EPIC1-s1) or ectopic Lnc-EPIC1 overexpression failed to alter functions of MYC KO colon cancer cells (Figure 5F–5H). Therefore, exogenously altering Lnc-EPIC1 expression was ineffective in MYC-KO colon cancer cells. These results indicate that Lnc-EPIC1-induced colon cancer cell progression is primarily due to association with MYC protein.

## DISCUSSION

Colon cancer is the third most prevalent cancer around the world [3, 4]. The vast majority of colon cancers develop from pre-existing adenomas, although some could also emerge *de novo* [18–20]. MYC (mainly c-MYC) is overexpressed in most human colon cancers, which is associated with carcinogenesis, tumorigenesis and progression [21–24]. Downregulation of MYC will induce cancer cell quiescent and apoptosis [21–24]. MYC is a multifunctional gene regulating cell division, growth, and apoptosis, and promoting the expression of several key oncogenes [21–24]. The underlying mechanisms of MYC dysregulation contributing to colon cancer progression are still not fully resolved.

Emerging studies have proposed that LncRNAs could serve as biomarkers of colon cancer for prognosis, diagnosis, even prediction of therapeutic results. These non-coding RNAs act as oncogenes or tumor suppressors, essential in the regulation of cancer cell behaviors, including proliferation, migration, survival, and apoptosis resistance [25]. Using the LncRNA epigenetic landscape analyses, Wang et al.*,* identified Lnc-EPIC1 as an oncogenic LncRNA that directly binds MYC protein [13]. Lnc-EPIC1 is overexpressed in human breast cancer [13]. Lnc-EPIC1 silencing resulted in decreased expression of MYC targeted genes and inhibited tumorigenesis *in vitro* and *in vivo* [13]. Lv et al.*,* demonstrated that Lnc-EPIC1-MYC association promoted cholangiocarcinoma cell growth [16]. Zhang et al.*,* found that Lnc-EPIC1-MYC protein binding is essential for lung cancer cell growth, survival and proliferation [15]. Recent studies proposed that Lnc-EPIC1 is expressed in human osteoblasts. Its downregulation can promote osteoblast cell death [17, 26].

The results of this study show that Lnc-EPIC1 is important for the progression of colon cancer cells. Lnc-EPIC1 expression is significantly elevated in human colon cancer tissues and primary colon cancer cells. Whereas low expression is detected in colon mucosa epithelial tissues and colon epithelial cells. In the primary colon cancer cells Lnc-EPIC1 siRNA largely inhibited cancer cell growth, proliferation, migration and invasion, while inducing cell death and apoptosis. Conversely, forced overexpression of Lnc-EPIC1, using a lentiviral construct, further promoted primary colon cancer cell progression *in vitro*. These results clearly indicate that overexpressed Lnc-EPIC1 is an important contributor of colon cancer cell progression. It could be valuable therapeutic target.

In this study, RIP and RNA pull down assay results confirmed that Lnc-EPIC1 directly binds MYC protein in the primary human colon cancer cells, essential for MYC’s function. Lnc-EPIC1 siRNA resulted in downregulation of MYC targets, including cyclin A, cyclin D and CDK9 in colon cancer cells. Conversely, their expression was elevated with forced Lnc-EPIC1 overexpression. To further support Lnc-EPIC1-mediated colon cancer cell progression is due to MYC association, we show that CRISPR/Cas9-inudced MYC KO mimicked Lnc-EPIC1 silencing-induced activity and inhibited colon cancer cell survival, proliferation and migration. More importantly, in MYC-KO colon cancer cells Lnc-EPIC1 siRNA or overexpression was completely ineffective on cell behaviors. These results clearly demonstrated that Lnc-EPIC1-mediated colon cancer cell progression is through binding to MYC protein.

Wang et al.*,* have reported that inhibition of LncRNA EPIC1-MYC association suppressed luminal breast cancer tumorigenesis *in vivo* [13]. Further studies will be needed to explore whether LncRNA EPIC1 expression and its association with MYC are important for the growth of colon cancer cell *in vivo*, for example using a mouse subcutaneous colon cancer model.

## MATERIALS AND METHODS

### Chemicals and reagents

Cell Counting Kit-8 (CCK-8) dye, JC-1 mitochondrial dye and the Annexin V apoptosis kit were purchased from Dojindo (Shanghai, China). Antibodies of cleaved-caspase-3, cleaved-caspase-9, cleaved-poly (ADP-ribose) polymerase (PARP), tubulin as well as MYC and its target genes were obtained from Cell Signaling Technology (Beverly, MA, USA). Cell culture reagents were provided by Gibco-BRL Co. (Gaithersburg, MD, USA). All the primers were synthesized by Shanghai Genechem Co. (Shanghai, China).

### Cell culture

NCM460 colon mucosa epithelial cells were purchased from the Cell Bank of Shanghai Institute of Biological Science, CAS (Shanghai, China). The primary human colon cancer cells, derived from three different written-informed human patients (“pri-Can-1/-2/-3”), were provided and verified by Dr. Xu at Tong-ren Hospital (Shanghai, China) [14]. The primary colon cancer cells were cultured in RPMI-1640 medium with 12% fetal bovine serum (FBS) and described supplements [27, 28]. The primary human colon epithelial cells were provided by Dr. Lu [27, 29, 30]. Primary colon cancer cells and epithelial cells at passage 3-10 were utilized for the experiments. The protocols of this study were approved by the Ethics Committee of Tongren Hospital, Shanghai Jiao Tong University School of Medicine, in accordance with the principle of Declaration of Helsinki.

### Human tissues

The primary colon cancer tissues and matched adjacent normal colon mucosa tissues (3-cm distant from the edge of original tumor) were obtained from a set of 10 primary written-inform consent colon cancer patients (administrated at Tongren Hospital, Shanghai Jiao Tong University School of Medicine). The patients received no chemotherapy (including targeted therapy) nor radiotherapy prior the surgery. Two independent pathologists confirmed colon cancer diagnosis.

### RNA extraction and quantitative real-time PCR (qPCR)

TRIzol reagents (Thermo-Fisher Invitrogen, Shanghai, China) was applied to extract total RNA from cultured cells and fresh human tissues. For each treatment, 2 μg of total RNA was reversely transcribed into complementary DNA (cDNA) via the PrimeScript RT kit (TaKaRa Biotechnology Co., Shanghai, China). An ABI7500 qPCR system (Oyster Bay, NY, USA) was utilized to conduct qPCR using an SYBR®Premix Ex TaqTM II kit (TaKaRa). The relative gene expression was calculated by a 2^−ΔΔCt^ method, with GAPDH tested as a control. The primers for testing Lnc-EPIC1, *liner EPIC1* and U6 were provided by Dr. Sun at Yangzhou University Medical School [17]. Lnc-EPIC1 expression was normalized to *U6 RNA*.

### Western blotting

Total cellular proteins were extracted using RIPA lysis buffer (Beyotime Biotechnology, Wuxi, China), with protein concentration determined by the BCA protein assay kit (Beyotime Biotechnology). For each treatment, equal amounts of protein lysates (20 μg) were separated by 10-12% SDS-PAGE gels and were then transferred to the polyvinylidene fluoride (PVDF) membranes (Millipore, Shanghai, China). After blocking using PBST with 10% non-fat milk, the blots were incubated with the applied primary detection antibodies at 4°C overnight. After incubation with secondary antibodies, the ECL detection reagents (Millipore, Shanghai, China) were utilized to detect the protein signals.

### Lnc-EPIC1 siRNA

As reported [15, 16], two different Lnc-EPIC1 siRNAs, 5’-CCUUCAGACUGUCUUUGAA-3’ (“si-Lnc-EPIC1-s1”) and 5’-GCUUUCUCUCGGAAACGUG-3’ (“si-Lnc-EPIC1-s1”), were synthesized by Shanghai Genechem Co. (Shanghai, China). Cells were seeded into the six well tissue culture plates at 50-60% confluence and were transfected with Lnc-EPIC1 siRNA or the non-sense scramble control siRNA (“scr” [17]) (each at 500 nM for 48h). The transfection was performed by using Lipofectamine RNAiMAX Reagent (Thermo-Fisher Invitrogen, Shanghai, China). Knockdown of Lnc-EPIC1 was verified by qPCR.

### Lnc-EPIC1 overexpression

Colon cancer cells were seeded into six well plates at 50-60% confluence, and cultured in polybrene-containing complete medium. Cells then were tranduced with a pLenti6-GFP-puro Lnc-EPIC1 expression vector (provided by Dr. Sun [17]) through Lipofectamine 2000. After 48h puromycin (5.0 μg/mL) was utilized to select stable cells, and two stable cell lines, OE-Lnc-EPIC1-Line1/2, were established. Lnc-EPIC1 overexpression was verified by qPCR. The empty vector (“Vec”, provided by Dr. Sun [17]) was transduced to the control colon cancer cells.

### Cell viability detection

Colon cancer cells with applied genetic modifications were plated in 96-well plates at 4,000 cells per well. Cells were further incubated for 96h. Afterwards, 20 μL of CCK-8 reagent was added into each well. CCK-8 absorption was detected at 450 nm on a microplate reader (BioTek, Winooski, VT, USA).

### EdU (5-ethynyl-20-deoxyuridine) assay of cell proliferation

The primary human colon cancer cells, with applied genetic modifications, were seeded at 1 × 10^5^ cells per well into the six-well plates. Cells were allowed to growth for 72h. Next, an EdU Apollo-567 Kit (RiboBio, Guangzhou, China) [28] was applied to examine cell proliferation. EdU and DAPI staining were visualized under a fluorescent microscope.

### Cell migration and invasion assays

Transwell chambers (12-μm pore size) were purchased from Corning Costar (Shanghai, China). The colon cancer cells (0.6 ×10^5^ cells per treatment, in serum free medium) with applied genetic modifications were placed onto the upper compartments of the transwell chambers. Complete medium with 12% FBS was added to the lower chambers [31]. After incubation at 37°C for 16h, cells that migrated through the membrane were fixed and stained. For the cell invasion assays, the apical chambers were coated with “Matrigel” [32].

### Flow cytometry assay of cell apoptosis

Colon cancer cells with the applied genetic modifications were cultured for 48h, washed with PBS, and trypsinized to get the single-cell suspensions. Cells were then fixed (in ice-cold 70% ethanol) and incubated with Annexin-V FITC and propidium iodide (PI) solution (each at 10 μg/mL, Sigma-Aldrich). FACS was performed on a BD FACSCalibur instrument (Shanghai, China), with Annexin V-positive cells gated and recorded.

### TUNEL staining

The TUNEL assay kit (KeyGEN BioTECH, Jiangsu, China) was utilized to examine and quantify cell apoptosis. In brief, colon cancer cells with applied genetic treatments were cultured for 48h and incubated with TUNEL mixture for 60 min. TUNEL staining was detected under a confocal microscopy.

### Caspase-3 activity assay

The primary human colon cancer cells, with applied genetic modifications, were seeded at 1 × 10^5^ cells per well into the six-well plates and cultured for 48h. Testing the caspase-3 activity was described early [33]. In short, 20 μg of cytosolic extract lysates per treatment were added to the caspase assay buffer [33] together with the 7-amido-4-(trifluoromethyl)-coumarin (AFC)-conjugated caspase-3 substrate [33]. AFC was measured through the Fluoroskan system [33].

### Mitochondrial depolarization

In cells with mitochondrial depolarization, JC-1 dye will aggregate into the inner membrane of mitochondria, forming green monomers [34]. The primary human colon cancer cells, with applied genetic modifications, were seeded at 1 × 10^5^ cells per well into the six-well plates. Cells were incubated with JC-1 (7.5 μg/mL) for 30 min under the dark, washed and tested immediately under a fluorescence spectrofluorometer at 488 nm. The representative merged JC-1 images, integrating both green and red fluorescence channels, were presented as well.

### RNA-immunoprecipitation (RIP)

Human colon cancer cells were trypsinized, washed, and incubated with 0.3% formaldehyde and glycine (0.125 M), RIP lysis buffer [13] was added to achieve total cellular lysates. Lysates (800 μg protein per treatment) were incubated with the anti-MYC antibody (Santa Cruz Biotech, Santa Cruz, CA, USA) overnight. MYC-bound pellets were washed, and re-suspended and incubated with the proteinase K-containing buffer. A qPCR assay was then performed to test MYC-bound RNA.

### RNA pull-down

Biotin-labeled full-length Lnc-EPIC1 was synthesized, sequence-verified and provided by Shanghai Genechem Co. (Shanghai, China). It was dissolved in RNA structure buffer and folded, put on ice immediately, and then transferred to room temperature [35]. For each treatment, 200 μg cleared nuclei lysates of cultured primary human colon cancer cells were mixed with folded Lnc-EPIC1 and Dynabeads MyOne Streptavidin C1 magnetic beads (“Beads”, provided by Dr. Wang at Soochow University [35]). Beads were washed, with the retrieved proteins tested by Western blotting.

### MYC knockout

The primary human colon cancer cells were seeded into the six well tissue culture plates at 50-60% confluence. Cells were transfected with applied lenti-CRISPR-MYC-KO-GFP construct (two constructs with different sgRNAs, provided by Dr. Sun at Yangzhou University Medical School [17]). The CRISPR GFP-positive cells were FACS sorted, followed by genotyping of depleted MYC. Two stable cell lines, MYC-KO-1 and MYC-KO-2 were established. Control cells were transfected with lenti-CRISPR-Cas9 empty vector (“Cas-C”).

### Statistics analyses

The statistical analysis was performed by the SPSS software (23.0, SPSS, Inc., Chicago, IL, USA) using the one-way analysis of variance (ANOVA) with Dunnett’s post hoc test. For comparing significance between two treatment groups a two-tailed unpaired T test was applied (Excel 2007). Experiments in this figure were repeated five times. *P* <0.05 was considered to statistically significant.
